# Measuring the Rheological and Textural Properties of Thick Purees Used to Manage Patients with Swallowing Disorders

**DOI:** 10.3390/nu15173767

**Published:** 2023-08-28

**Authors:** Kovan Ismael-Mohammed, Mireia Bolivar-Prados, Laura Laguna, Pere Clavé

**Affiliations:** 1 Gastrointestinal Physiology Laboratory, Hospital de Mataró, Universitat Autònoma de Barcelona, 08304 Mataro, Spainmbolivar@csdm.cat (M.B.-P.); 2 Institute of Agrochemistry and Food Technology (IATA-CISC), 46980 Valencia, Spain; laura.laguna@iata.csic.es; 3 Centro de Investigación Biomédica en Red de Enfermedades Hepáticas y Digestivas (Ciberehd), 08304 Barcelona, Spain

**Keywords:** dysphagia, viscosity, textural properties, rheometer, texture analyzer, texture C, thick puree

## Abstract

Texture-modified diets are the first-line compensatory strategy for older patients with swallowing and mastication disorders. However, the absence of a common protocol to assess textural properties inhibits their standardization and quality control and, thus, patient safety. This study aimed to (a) assess the rheological and textural properties of ten thick purees (Texture C, British Dietetic Association), (b) understand the effect of oral processing, and (c) measure the properties of the ready-to-swallow bolus after oral processing in healthy adults. Shear viscosity at 50 s^−1^ and 300 s^−1^ and textural properties (maximum force, cohesiveness, and adhesiveness) of boluses of ten thick purees were analyzed with a rheometer and a texture analyzer before and after oral processing (ready-to-swallow) in five healthy volunteers. Viscosity varied by 81.78% at 50 s^−1^ (900–4800 mPa·s) among purees before oral processing. Maximum force varied by 60% (0.47–1.2 N); cohesiveness, 18% (0.66–0.82), and adhesiveness, 32% (0.74–1.1 N·s). The high variability of viscosity was also present in ready-to-swallow boluses, 70.32% among purees. Oral processing significantly reduced viscosity in most purees (French omelet, zucchini omelet, turkey stew, red lentils, noodles, and hake fish) and also significantly reduced maximum force (7–36%) and adhesiveness (17–51%) but hardly affected cohesiveness (<5%). All thick purees met the qualitative textural descriptors for Level C texture. However, all ten purees showed significant differences in all parameters measured instrumentally and were affected differently by oral processing. This study demonstrates the need to use instrumental quality control using standardized protocols and SI units to narrow the variability and provide the optimal values for patients with dysphagia who require texture-modified diets.

## 1. Introduction

As we age, our bodies undergo changes that can impact our physical and cognitive abilities [1,2]. Mastication and swallowing are two processes strongly affected by aging [3,4,5], which can be aggravated by disorders such as oropharyngeal dysphagia (OD) [6,7]. These impairments not only have an impact on health (including malnutrition, dehydration, and aspiration pneumonia) but also on patients’ quality of life [8,9].

The prevalence of swallowing disorders in older individuals can range from 10% to 33% [10]. Another study found that the prevalence of OD in independently living older individuals was 16.6% for those aged between 70 and 79 and 33% for those over 80. The study also showed that 47% of hospitalized older people had OD [11]. Likewise, a large proportion of the older population has mastication impairments [12]. Dias-da-Costa’s (2010) study in Brazil discovered that 49.7% of participants reported poor mastication [13]. In Mexico, a study carried out by Murrieta et al. (2016) showed a 33.1% prevalence of temporomandibular disorders among people aged 60–69 years old [14]. Dibello et al. (2021) found reduced oral motor skills, including masticatory function (9%), as contributing factors to frailty in older adults, particularly those with few remaining teeth (29%) and mastication difficulties (11%) [12].

Shear viscosity has a major role in the therapeutic effect produced by thickened fluids (TF) in patients with oropharyngeal dysphagia [15,16,17]. The increment in shear viscosity increases the prevalence of safe swallowing in all phenotypes of patients [15,17]. However, scientific evidence is needed to determine and prescribe the viscosity levels that have the greatest therapeutic effect and describe them in the International System of Units (SI). Thickened fluids have a non-Newtonian behavior (viscosity decreases with the increment of shear rate, called shear thinning). In addition, depending on their composition, viscosity can also be reduced by alpha salivary amylase during the oral preparatory phase [15,18]. Our research team has designed a protocol to assess the viscosity of TF used to treat OD considering the rheological factors associated with the oral phase (including salivary amylase) and pharyngeal phase (shear thinning associated with pharyngeal bolus flow) of swallowing [19]. An ex vivo experiment was performed to assess the effect of oral salivary amylase, where participants held a 15 mL bolus in their mouth for 30 s and then spat it out for measurement. The effect of shear rate was measured in an in vitro study across the whole swallowing spectrum of oropharyngeal shear rate (1–1000 s^−1^), where viscosity values at 50 s^−1^ (oral cavity) and 300 s^−1^ (pharyngeal phase) were interpolated [18].

The use of qualitative descriptors to classify the thickness of alimentary fluids, such as “thin” or “thick”, versus SI units of viscosity and texture measurement of alimentary fluids and semisolid food is a subject under discussion in the field of food hydrocolloids [20]. There is much variation in the qualitative descriptors used by different classification systems for the same viscosity level, and the viscosity levels between different systems vary a lot. The National Dysphagia Diet (NDD) uses the descriptors Thin (<50 mPa·s), Nectar-thick (51–350 mPa·s), Honey-thick (351–1750 mPa·s), and Spoon-thick (>1750 mPa·s) [21]. In contrast, In Japan, two systems are used to classify alimentary fluids: the Japanese Dysphagia Diet (JDD) 2021 for fluids and the Smile care system for solid food. The JDD divides fluids into three levels: Mildly thick (50–150 mPa·s), Moderately thick (150–300 mPa·s), and Extremely thick (300–500 mPa·s), and the Smile Care classifies them based on the ability of the people for mastication and swallowing [22].

Nowadays, there is no standardized protocol to determine the optimal texture of texture-modified diets for patients with mastication and swallowing disorders. The majority of existing food classifications are subjective and based on qualitative descriptors such as the British Dietetic Association (BDA), which classifies texture-modified diets into four categories: B (thin puree), C (thick puree), D (pre-mashed diet), and E (fork-mashable diet) [23]. More recently, the International Dysphagia Diet Standardisation Initiative (IDDSI) developed a classification for alimentary liquid and solid food of eight levels (0–7) using qualitative descriptors and empirical measurement methods. The IDDSI flow test (using a syringe) is used to classify five levels of liquids (0–4), while the fork pressure test characterizes five levels of diets (3–7) [24]. For texture-modified foods, the Smile care system categorizes food according to a person’s ability to masticate: food for healthy people (blue), for those with mastication impairments (yellow), and for those with swallowing impairments (red) [25]. The Triple Adaptation of the Mediterranean diet has adapted 296 Mediterranean dishes according to the BDA classification and descriptors for C-texture (thick puree) and E-texture (fork-mashable) [26]. The therapeutic effect of these diets has been recently assessed in a clinical trial evaluating a Minimal-Massive Intervention for older patients with OD, which also considered the process of oral processing, defined as the process of breaking down and mixing food with saliva in the mouth before swallowing [27].

The Texture Profile Analysis (TPA) protocol uses specialized equipment (texture analyzer) to mimic the oral processing of food, aiming to identify textural characteristics from the first two bites. It was introduced by Friedman, Whitney, and Szczesniak in 1963 and is popular in the food industry due to its simplicity and ability to measure food textures comparable to those experienced during oral processing. During this process, food is mechanically broken down by the teeth and tongue and degraded by enzymes in saliva [28]. Oral processing can be studied both in vitro and in vivo. In vitro studies simulate the mechanical and enzymatic processes of chewing and digestion using mechanical devices to grind and mix food with artificial saliva. In vivo studies involve observing and measuring muscular activity and organ function in humans or animals while consuming food [19]. Most studies on oral processing have been conducted on solid foods, as they require more chewing and processing activity to be properly digested. Some examples of foods that have been studied include meat, raw fruits and vegetables, bread, crackers, and cereal [29]. Recently, we have designed and validated a rheological protocol to reproduce the oral processing and pharyngeal factors that affect the therapeutic effect of alimentary fluids and report the viscosity measurements in a standardized, scientific, and precise manner [19].

By establishing objective criteria to determine the appropriate consistency of modified diets, individuals with swallowing and mastication impairments can receive the safest and most appropriate nutrition and support tailored to their swallowing function. Therefore, the main objective of this study was to assess the rheological (viscosity, mPa·s) and textural properties (maximum force N, cohesiveness, and adhesiveness N·s) of ten thick purees (Texture C, BDA) in a hospital setting destined for patients with swallowing and mastication disorders, and to understand the effect of oral processing (OP) and the properties of the ready-to-swallow bolus with healthy volunteers.

## 2. Materials and Methods

### 2.1. Materials

#### 2.1.1. Texture-Modified Diets (TMDs)

Ten texture-modified thick purees from the Mediterranean diet [19] were selected and provided by Catering Arcasa SL (Esplugues de Llobregat, Spain). These thick purees were Level C texture (thick purees) according to BDA and included: A-French omelet, B-zucchini omelet, C-pumpkin, D-stewed turkey, E-pollock fish, F-red lentils, G-noodles, H-hake, I-cauliflower, and J-broccoli.

#### 2.1.2. Thick Purees Formulation

Thick purees were prepared in the hospital kitchen to meet the qualitative descriptors for Level C texture. Table 1 presents the characteristics and texture check for this texture.

#### 2.1.3. Thick Puree Composition

Table 2 provides an estimation of the thick puree composition, providing information on ingredients and proportions. The table includes information on an estimation of the ingredient’s percentage, carbohydrates, protein weight in g, and kilocalories for all the thick puree tested. Images of the chosen Mediterranean thick purees for this study are displayed in Figure S1 (see Supplementary Material S1).

#### 2.1.4. Participants

Five healthy adults (three women and two men, 30 ± 3.9 years of age) participated in this study. The study inclusion criteria were to be between 18 and 40 years, to be mentally able to sign the informed consent to participate, and to have normal mastication and swallowing function, with no swallowing or mastication impairments. The exclusion criteria were not meeting the inclusion criteria or not being able to try all the 10 thick purees due to allergies and having Sjögren syndrome or sialorrhea.

### 2.2. Study Design

**Figure 1:**
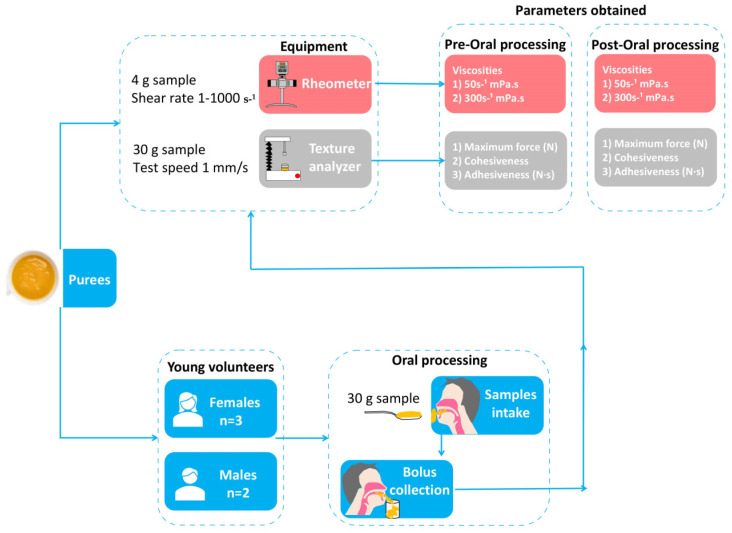
Scheme of the study design including the two protocols for rheological and textural properties of each thick puree assessed at pre- and post-oral processing.

This study was divided into two main steps, as illustrated in Figure 1. The first step involved pre-oral processing, in which the rheological and textural properties of the thick purees were analyzed. In the second step, post-oral processing, all thick purees were subjected to oral processing by participants. Participants were asked to hold 30 g of each puree in their mouths, process it as they normally would, and spit it out when they felt the bolus was ready to swallow. The rheological and textural properties of the collected bolus were then analyzed. The procedure was approved by the Ethics Committee of the Consorci Sanitari del Maresme, codified under 63/22.

### 2.3. Methodology

Three different batches of each thick puree were selected for analysis and taken to the laboratory separately, maintaining their temperature.

#### 2.3.1. Rheological Properties

Four grams of each texture-modified diet was placed in the CC27/QC-LTD sensory system of the Anton Paar RheolabQC rheometer (Graz, Austria), and data were extracted using RheoCompass (version 1.30. Ink) software. Shear rates ranging from 1 to 1000 s^−1^ were used, and viscosity values calculated were at shear rates of 50 s^−1^ (simulating oral shear rate) and 300 s^−1^ (simulating pharyngeal shear rate). The temperature was set at 40–50 °C for pre-oral processing and 25 °C for post-oral processing. Shear viscosity values were expressed in SI units (mPa·s). The Ostwald–de Waele equation (also known as the Power Law Model) [18] was used to check the flow and consistency indexes and describe the non-Newtonian behavior of thick purees. The following equation was used:

σ = Kγ^n^

where σ is the shear stress (Pa), γ is the shear rate (s^−1^), and K, referred to as the consistency index, is measured in units of Pa sn^(n−1)^, where n represents the flow index. The consistency index is a dimensionless value that provides a measure of the consistency of a non-Newtonian fluid. On the other hand, the flow index (also known as the power-law index or Ostwald–de Waele exponent) is denoted by the symbol “n”. It ranges between 0 and 1 and is used to classify the fluid into different categories. For instance, a fluid with n = 1 is classified as a Newtonian fluid, while a fluid with 0 < n < 1 is considered pseudoplastic. On the contrary, a fluid with n > 1 falls under the category of dilatant fluids.

#### 2.3.2. Textural Properties

All ten thick purees were analyzed using a Texture Profile Analysis (TPA) [30] using the TA.XTPlus Texture Analyzer (Stable Micro Systems, Godalming, UK). Data collection and management were carried out with the exponent software (version 7.0.6.0 Stable Microsystems). The texture analyzer cell was loaded with a force of 5 kg and a trigger force of 0.049 N. The probe used was an aluminum cylinder probe with a diameter of 36 mm, which was paired with an acrylic recipient (internal diameter of 49.5 mm and height of 72 mm). A texture profile analysis test was performed on each sample by introducing 30 g of the puree into the acrylic recipient, and it was measured at a temperature between 40 and 50 °C. The test speed was set at 1 mm/s. From the force-displacement curve, the maximum force taken from the first peak force was considered as the maximum force, cohesiveness was calculated as the ratio between the area of the second peak and the area of the first peak, and adhesiveness was calculated as the first negative area. Values from TPA were expressed in SI units for the three textural parameters assessed.

#### 2.3.3. Saliva and Oral-Processing Effect Calculation

The effects of saliva and oral processing on both viscosity and textural characteristics (maximum force, cohesiveness, and adhesiveness) were calculated and expressed in SI units. The values obtained during pre-oral processing were used as a reference to measure the effects of oral processing measured by the rheometer and those measured by the texture analyzer using the following formulas [20]:

- Viscosity decay was assessed according to the following formula:

[(viscosity at 50 s^−1^ − viscosity at 50 s^−1^ after oral processing/viscosity at 50 s^−1^] × 100

- The shear thinning effect on the viscosity during pharyngeal flow decay in viscosity due to shear rate was assessed according to the following formula:

[(viscosity at 50 s^−1^ − viscosity at 300 s^−1^/viscosity at 50 s^−1^] × 100

- Viscosity decay due to the combined effect of both shear thinning and the effect of oral processing decrease in the viscosity due to the shear rate was assessed according to the following formula:

[(viscosity at 50 s^−1^ − viscosity at 300 s^−1^ after oral processing/viscosity at 50 s^−1^] × 100

- Maximum force cohesiveness and adhesiveness decay were assessed according to the following formula:

[(maximum force before oral processing − maximum force after oral processing/maxi mum force before oral processing] × 100

### 2.4. Data Management and Statistical Analysis

T-score was used to remove the outliers according to the following formula, where the range analyzed was 30 to 70 in terms of T-score.

T = 50 + (10 (X − Mean)/SD),

Each puree was analyzed three times per day on five separate days. The variability of viscosity and textural properties among the thick purees before and after oral processing was assessed using a one-way analysis. To determine the least significant differences, the Tukey test was employed, and a significance level of *p* < 0.05 was adopted. Additionally, the Mann–Whitney nonparametric test was used to detect significant distinctions between pre- and post-oral processing concerning viscosity and textural properties. To check the differences between the pre and post-shear thinning percentage, a Z test was used. A Pearson correlation matrix was performed to see the correlation among the parameters. All analyses were conducted using XLSTAT 2020.4.1 (Addinsoft, Paris, France)

## 3. Results

### 3.1. Shear Viscosity at 50 s^−1^

#### 3.1.1. Pre-Oral Processing

The viscosity results for all the thick purees at 50 s^−1^ are shown in Table 3. The apparent viscosity presented significant differences (*p* < 0.0001) among thick purees, the thick cauliflower puree being the thinnest (875.97 ± 128.2 mPa·s), and the noodle thick puree, the thickest (4809.85 ± 1618.21 mPa·s).

#### 3.1.2. Oral-Processing Effect

Table 3 includes the effect of oral processing and saliva amylase on viscosity measurements at a shear rate of 50 s^−1^ for pre- and post-oral processing. It was found that saliva and oral processing reduced the viscosity of the thick purees, with a reduction in viscosity ranging from 1.5% to 71%. Two main patterns were observed for the effect of oral processing on the shear rate of 50 s^−1^: (1) oral processing-resistant thick purees (20%; cauliflower and broccoli) were not affected by oral processing, with a non-significant reduction of 1.5–7%; and (2) oral processing-sensitive thick purees (80%) were affected by oral processing (9–71%, *p* < 0.05).

#### 3.1.3. Ready-to-Swallow Bolus

The results of the study, as shown in Table 3, show that the apparent viscosity of the ready-to-swallow bolus after oral processing of the 10 thick purees was slightly variable. The thick puree with the highest viscosity was the noodle bolus, with a mean value of (1872.71 ± 944.2 mPa·s), while the thick puree with the lowest viscosity was the French omelet bolus, with a mean value of (555.73 ± 356.50 mPa·s). Statistical analysis showed significant differences (*p* < 0.05) in viscosity among the different thick purees (see Table 3). The variability in the apparent viscosity in mPa·s of the ready-to-swallow boluses for the 10 thick purees that met the qualitative descriptors for Texture C (Thick Puree Dysphagia Diet) was 70.32% (see Table 3).

#### 3.1.4. Pre-Oral Shear Thinning and Combined Effect with Oral-Processing Effect

The results found that the thick purees had high levels of shear thinning, both pre- and post-oral processing, as presented in Table 4. The measurements showed 72–79% shear thinning pre-oral processing, similar to the 66–78% post-oral processing; no significance was identified except for when shear thinning of pre- compared to post- for noodles. The combined effect of oral processing and shear thinning during pharyngeal flow caused a significant decrease in viscosity, with a range of 67–93%. Additionally, viscosity at 300 s^−1^ was found to be lower post-oral processing compared to pre, except for pollock fish, cauliflower, and broccoli. The study also found a statistical difference in shear thinning when comparing pre-oral and post-oral processing for noodles, with a *p*-value < 0.0001.

#### 3.1.5. Effect of Oral Processing on Non-Newtonian Behavior

The flow index (n) of the thick purees at pre-oral processing ranged from 0.22 for the pollock fish thick puree to 0.87 for the red lentils thick puree, and post-oral processing ranged from 0.06 for the red lentils thick puree to 0.93 for the zucchini omelet thick puree. A non-significant slight change in the flow index (n) was observed in the thick purees of French omelet, zucchini omelet, stewed turkey, and noodles after oral processing, and the red lentils showed a significant reduction after the oral processing (Table 5). Similarly, the consistency index (K) of the thick purees before oral processing ranged from 3.27 Pa·s^n^ for the red lentil thick puree to 4.78 Pa·s^n^ for the noodle thick puree, and after oral processing ranged from 3.98 Pa·s^n^ for the French omelet to 4.82 Pa·s^n^ for the red lentil. A slight variation in the consistency index was observed in the thick purees of French omelet, zucchini omelet, stewed turkey, red lentils, and noodles after oral processing (Table 5).

**Figure 2:**
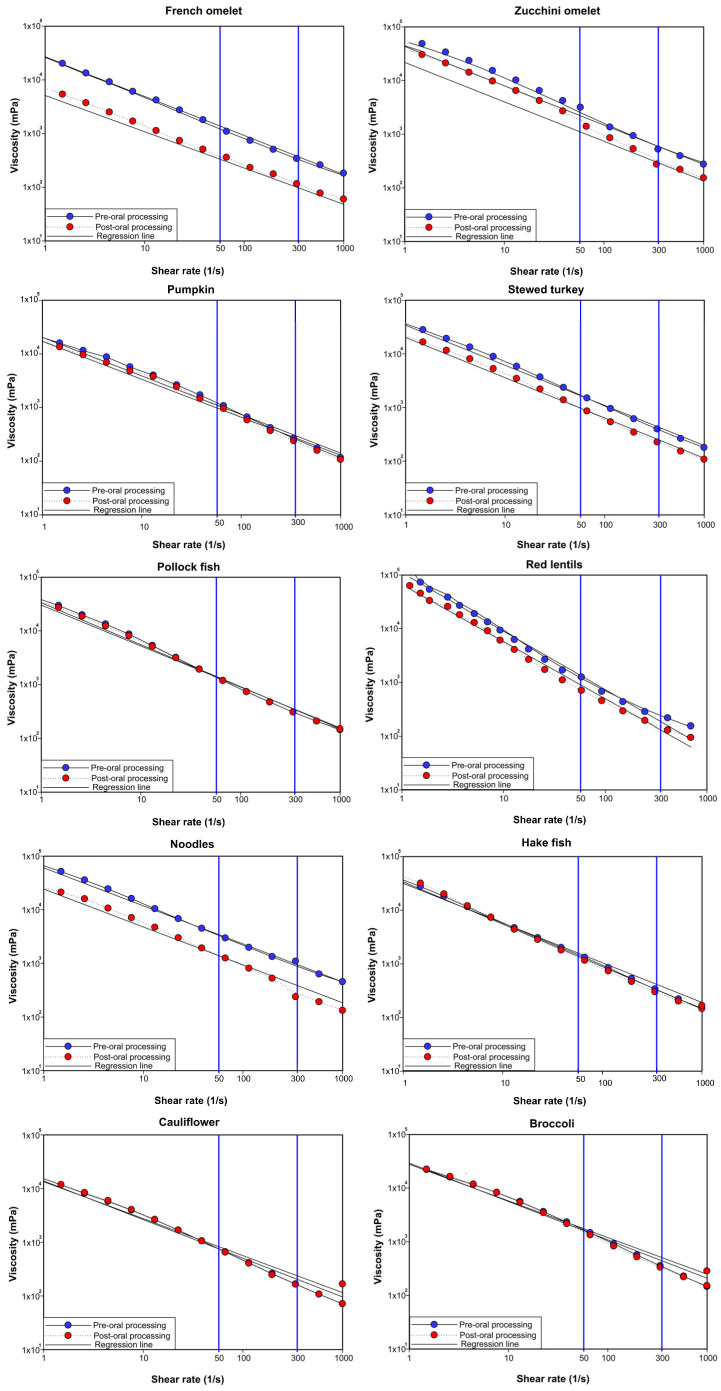
Mean viscosity curve graphs for each thick puree, pre- and post-oral processing, from 1 to 1000 s^−1^ shear rate, values at 50^−1^ and 300^−1^ are marked with blue lines.

Figure 2 shows the average viscosity of each puree before and after the oral processing. Focusing on the values corresponding to the shear rates of 50 s^−1^ and 300 s^−1^ of the ten thick purees tested.

**Figure 3:**
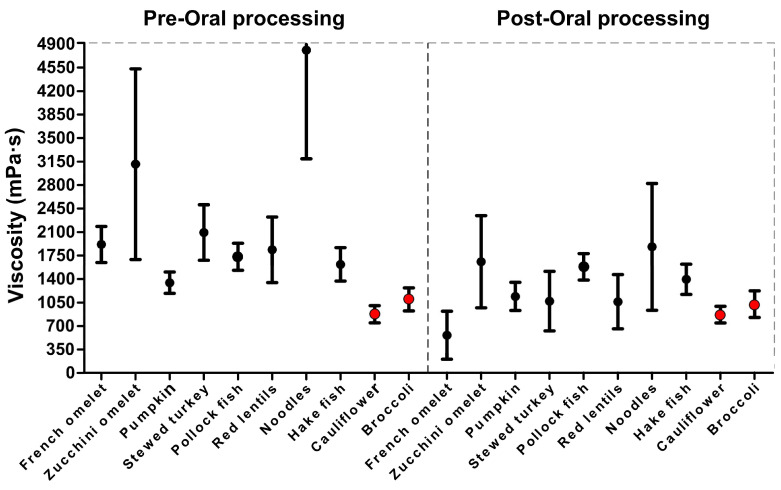
Viscosity (at 50 s^−1^) for the ten thick purees pre- and post-oral processing. Red points depict oral processing-resistant thick purees.

In this study, the viscosity of the thick puree was measured at a shear rate of 50 s^−1^, pre- and post-oral processing. The shear rate viscosity of all the thick purees at a shear rate of 50 s^−1^ for the pre-oral processing is shown in Figure 3.

### 3.2. Textural Characteristics

**Figure 4:**
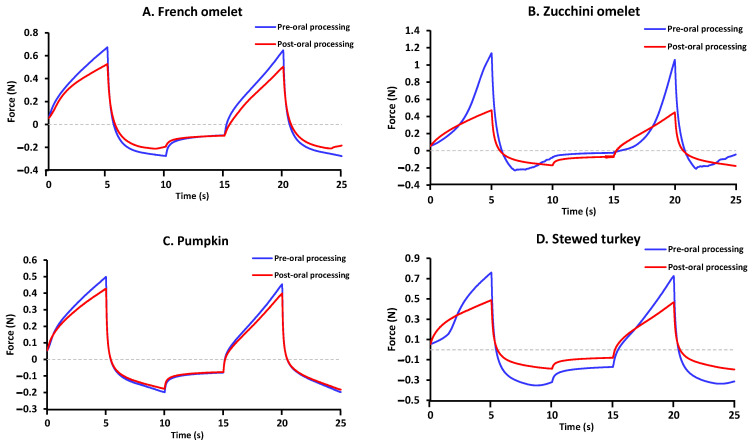

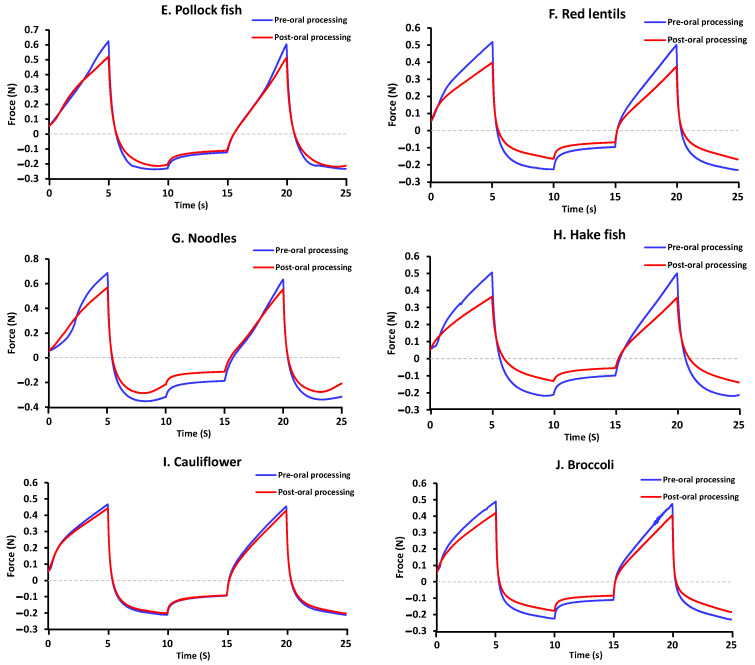
Examples of the curves obtained with the TPA test of the 10 thick purees pre- and post-oral processing ((**A**)_French omelet, (**B**)_zucchini omelet, (**C**)_pumpkin, (**D**)_stewed turkey, (**E**)_pollock fish, (**F**)_red lentils, (**G**)_noodles, (**H**)_hake, (**I**)_cauliflower, (**J**)_broccoli).

The texture profile analysis (TPA) graphs for pre- and post-oral processing are shown in Figure 4 for the ten thick purees.

#### 3.2.1. Pre-Oral Processing

The results of the maximum force for the ten thick purees tested are shown in Table 6. The zucchini omelet thick puree presented the highest maximum force (1.2 ± 0.85 N) and showed significant differences (*p* < 0.0001) when compared with the rest of the thick purees. Hake fish had the lowest maximum force (0.47 ± 0.03 N). The slight variation in cohesiveness is shown in Table 7, with hake fish being the most cohesive (0.82 ± 0.03), significantly more than the other purees (*p* < 0.0001). Finally, the most adhesive thick puree was French omelet (1.1 ± 0.23) but only significantly different (*p* < 0.05) when compared to the least adhesive thick puree of pumpkin (0.74 ± 0.21), Table 8.

#### 3.2.2. Oral-Processing Effect

Oral processing had a significant effect on the textural properties of thick purees. When compared with their pre-oral processing values, oral processing caused a decrease in maximum force ranging from 6.38 to 36.49% in 5 out of 10 thick purees (Table 6). The cohesiveness of the thick purees ranged between no effect and a 4.88% reduction, with significant differences in 4 out of 10 thick purees (Table 7), and adhesiveness was reduced by between 17.57 and 50.91% with significant differences (*p* < 0.05) in 6 out of 10 thick purees (Table 8). Oral processing did not cause any significant change in the three textural properties measured by TPA in 2 out of 10 thick purees (pumpkin and cauliflower).

#### 3.2.3. Ready-to-Swallow Bolus

The textural properties of the ready-to-swallow boluses were examined, and results are presented in (Table 6, Table 7 and Table 8). Significantly different results were observed among the ready-to-swallow boluses for maximum force, zucchini omelet having the largest maximum force (0.85 ± 0.13) and red lentils and cauliflower having the smallest (0.42 ± 0.07, *p* < 0.05). In terms of cohesiveness, there were significant differences (*p* < 0.05) among the bolus ready-to-swallow BRS, with the highest cohesiveness observed for the cauliflower and broccoli BRS (0.80 ± 0.03) and the lowest for the zucchini omelet BRS (0.70 ± 0.04). Furthermore, significant differences (*p* < 0.05) were also noted among the BRS for adhesiveness, with the highest adhesiveness observed for the zucchini omelet and the lowest for the stewed turkey.

#### 3.2.4. Textural Differences between Thick Purees Sensitive and Resistant to Oral Processing

Table 9 shows the mean values for viscosity at 50 s^−1^ were significantly higher at oral processing-sensitive purees (1287.24 mPa·s) when compared to oral processing-resistant purees (941.01 mPa·s; *p* = 0.001). The maximum force presented the same behavior (0.52 N compared to 0.48 N) (*p* = 0.02). However, the cohesiveness of the oral processing-resistant purees (0.77) was larger and more significant when compared to the oral processing-sensitive purees (0.74) (*p* = 0.0001), while adhesiveness did not show any significant differences between both oral processing-sensitive and oral processing residents 0.61 for oral processing-sensitive purees and 0.64 for oral processing-resistant purees (*p* = 0.6).

**Figure 5:**
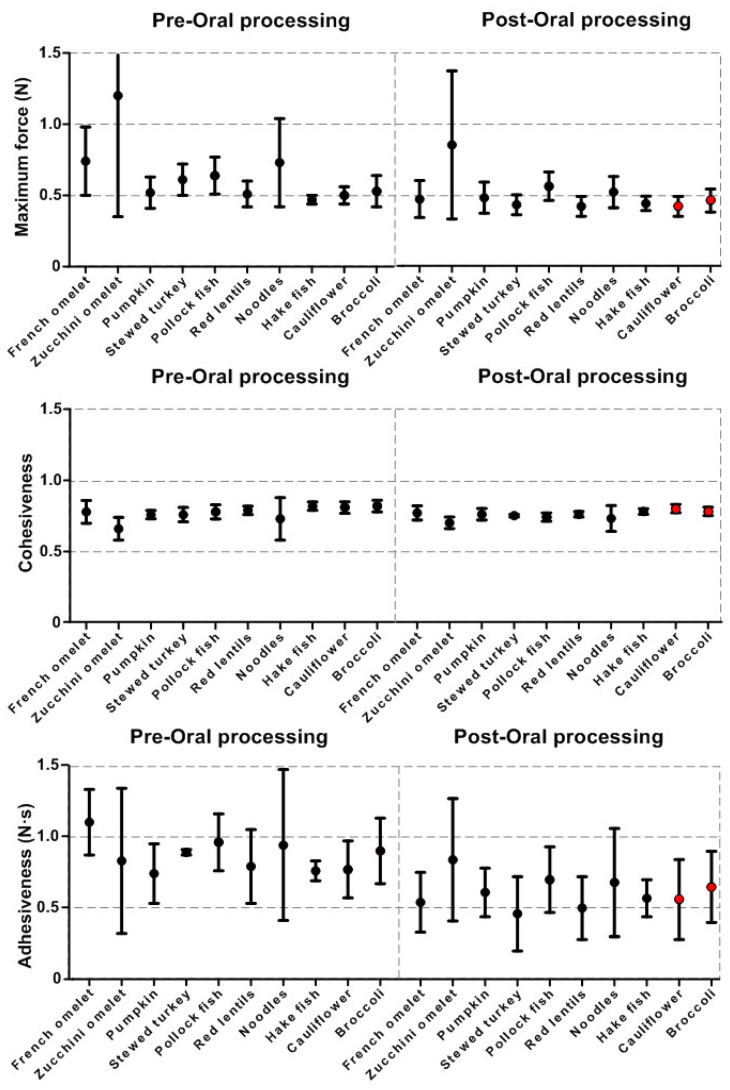
Maximum force, cohesiveness, and adhesiveness were plotted for all thick puree ranges for pre- and post-oral processing. Red points depict oral processing-resistant thick purees.

Figure 5 shows the mean values of the textural properties (maximum force, cohesiveness, and adhesiveness) for all thick puree ranges and how they change due to oral processing and are categorized into oral processing-resistant (red points) and oral processing-sensitive (black points).

### 3.3. Correlation Analysis

To see the correlation among the parameters tested, a Pearson correlation analysis was performed. In the pre-oral-processing variables (Table 10), we found a correlation between both viscosity points 50 s^−1^ and 300 s^−1^ was a low positive correlation (r = 0.49, *p* < 0.0001), and was a negligible correlation (r = 0.28, *p* = 0.0004) between maximum force and viscosity at a shear rate of 300 s^−1^. In addition, a moderate negative correlation between viscosity 300 s^−1^ and cohesiveness (r = −0.54, <0.0001) and adhesiveness was observed. Finally, a negligible correlation was also found between both cohesiveness and adhesiveness with (r = −0.29, 0.0003).

At post-oral processing (Table 11), a low-positive significant correlation was found between viscosity at 50 s^−1^, viscosity at 300 s^−1^ (r = 0.47, <0.0001), and cohesiveness (r = −0.42, <0.0001) while the correlation was negligible with maximum force (r = −0.29, 0.0003). A moderate positive correlation was found between cohesiveness and both maximum force and viscosity at 300 s^−1^ (r = −0.54, <0.0001) and maximum force with adhesiveness (r = −0.64, <0.0001).

## 4. Discussion

The present study investigated the viscosity and textural characteristics before and after oral processing of ten different thick purees designed for patients with mastication and/or swallowing impairments that met the qualitative textural descriptors for Level C texture according to the BDA descriptors. In this study, thick purees exhibited high viscosity values, ranging from 900 to 4800 mPa·s. Following oral processing, viscosity underwent a heterogeneous reduction varying from 1.5% to 71%. A remarkable shear thinning behavior (non-Newtonian) was also observed in all purees, with viscosity decreasing (72–80%) as shear rates representing pharyngeal bolus flow. The combination of both rheological factors (salivary amylase and dilution and shear thinning) reduced the initial viscosity by up to 80% in the pharyngeal stage. Regarding textural parameters, maximum force ranged from 0.47 to 1.2 N between the purees, cohesiveness between 0.66 and 0.82, and adhesiveness from 0.74 to 1.1 N. Oral processing also caused a heterogeneous reduction of all textural parameters (0–51%) with a small group of purees (2/10) unaffected during the oral phase. This study clearly shows that all thick purees prepared in a hospital kitchen met the qualitative textural descriptors for Level C texture but varied a lot in viscosity and texture, which affects their standardization. The high variability of viscosity and texture between purees was also observed in ready-to-swallow boluses. This study further demonstrates the need to use SI units and viscosity measurements to improve the confection of these thick purees to narrow the range of viscosities and textures and standardize and optimize the clinical management of the prevalent phenotype of hospitalized older patients with dysphagia.

The viscosity values of purees varied by 80%, while textural properties varied between 60%, 18%, and 32% for maximum force, cohesiveness, and adhesiveness, respectively. We have previously shown that qualitative descriptors such as those used by BDA, IDDSI, and NDD for thickened fluids are inaccurate and unscientific [19]. The lack of precision in qualitative texture classification also poses a considerable challenge to the development of consistent and reliable guidelines for texture modification of solid foods, especially on an industrial level. Therefore, there is a need to determine a textural protocol to analyze rheological and textural properties (viscosity, maximum force, cohesiveness, and adhesiveness) of purees and texture-modified diets in a scientific and accurate manner using quantitative measurements.

The main results of the ESSD White Paper on the Effect of Bolus Viscosity on the Safety and Efficacy of Swallowing in Patients with OD showed that increasing bolus viscosity results in (a) increased safety of swallowing, (b) increased amounts of oral and/or pharyngeal residue, which may result in post-swallow airway invasion, (c) reduced palatability of thickened fluids, and (d) recommended new randomized controlled trials to establish optimal viscosity levels for each phenotype of patients with dysphagia and that descriptors, terminology, and viscosity measurements must be standardized [16]. A recent study developed by our team [19] established a rheological protocol to measure the viscosity of thickened fluids for the hydration of dysphagic patients. Our present study demonstrates that the same protocol is also optimal for thick purees designed for optimal nutrition of patients with swallowing disorders and that measuring viscosity in SI units is mandatory to provide a safe swallow with these purees.

Optimal viscosity levels for patients with swallowing disorders have been determined in previous clinical trials and could be useful to guide the design of safe purees for dysphagic patients. We have extensively explored the effect of fluid thickening to provide safe hydration to older and neurological patients with OD [15,28,31]. For thin liquids with a viscosity of <50 mPa·s, at a shear rate of 50 s^−1^, the prevalence of safe swallowing was only 41.2%. However, with viscosities of 150, 250, and 450 mPa·s, the prevalence increased to 71.9–82.5%. Further increasing the viscosity to 800, 1400, and 2000 mPa·s resulted in even higher rates of safe swallowing, ranging from 91.2% to 95.6% at 800 and 1400 mPa·s with an XG thickening agent [15]. In terms of oral residues, thin liquids had a prevalence of 38.6% of patients and were also significantly increased with all thickened viscosity [15]. In another study by Ortega et al. (2020) [17], involving viscosities of <50, 250, 1000, and 2000 mPa·s of a mixed gum/starch thickener, the highest swallowing safety was observed at 1000–2000 mPa·s. The average safety rates were 82.47% and 95.88% for different patient phenotypes. Increasing the viscosity of thickeners (1000–2000 mPa·s) significantly increased oral residues but had no significant impact on pharyngeal residues in all phenotypes of patients. For older patients, oral residue prevalence was 86.11% and 72.22% at 1000 mPa·s and 2000 mPa·s, respectively. In patients with HNC, prevalence ranged from 66.67% to 64.52%. Parkinson’s patients showed a prevalence of 56.67% at both viscosities, while stroke patients had a prevalence of 50% and 54.84%. Oropharyngeal residue prevalence was 52.78% and 55.56% for older patients, 88.89% to 93.55% for HNC patients, 73.33% for Parkinson’s, and 66.67% to 61.29% for stroke patients [17]. In the present study, the viscosity values for purees ranged from 900 to 4800 mPa·s. However, previous studies have shown that viscosity above 2000 mPa·s does not further increase the safety of swallow and may even contribute to an increase in oropharyngeal residue. Adjusting the viscosity of thick purees within a range of 1000–2000 mPa·s would ensure a safe swallow with minimal residue, being a first significant step towards the standardization of thick purees for patients with oropharyngeal dysphagia.

Few studies have used Texture Profile Analysis (TPA) to assess the suitability of purees for individuals with swallowing difficulties (OD) [32]. Despite the weak to moderate significance of the correlations we found in our study, findings from other studies suggest that purees with lower firmness and adhesiveness values (below 2 × 10^4^ N/m^2^ and between 5 and 10 J/m^3^, respectively) and moderate cohesiveness values (0.3–0.4) are suitable for patients with OD. In this study, pre-oral processing showed that maximum force has a positive significant correlation with viscosity at a shear rate of 300 s^−1^, and cohesiveness and adhesiveness had a negative correlation. Post-oral processing, maximum force showed a positive correlation with both viscosities, cohesiveness, and adhesiveness. This highlights how these parameters correlate and how they are influenced by oral processing.

One of the key findings of this study is the identification of ready-to-swallow generated properties after oral processing. It was found that oral processing presented different effects on the reduction of viscosity, which led to two different viscosity patterns of those purees: oral processing-resistant purees that did not show any significant viscosity reduction (1.5–7%) and oral processing-sensitive purees that did (9–71%). In terms of texture parameters, oral processing led to reductions of 7–36%, <5%, and 17–51% for maximal force, cohesiveness, and adhesiveness, respectively. These findings highlight the considerable variation in viscosity and texture between purees before and after oral processing, with a range of 555–1575 mPa·s. While texture parameters were initially heterogeneous before oral processing, they became more consistent after oral processing, with narrower ranges observed: 0.42–0.85 (MF), 0.70–0.80 (C), and 0.46–0.84 (A). The information obtained from the ready-to-swallow boluses in healthy volunteers may have several applications for OD patients. Boluses can be customized based on viscosity reduction due to oral processing, which can make boluses more suitable for patients with OD. The information on texture could also induce puree adaptations for patients with bolus formation difficulties.

The main weaknesses of this study are that we only included healthy volunteers, limiting the finding to the targeted group, and did not add the sensory aspect for puree preferences. Further studies on older patients with OD should be performed to progress in the design of safe, efficient, nutritive, and tasty purees for older patients with swallowing disorders.

## 5. Conclusions

In conclusion, this study highlights the importance of standardizing the measurements of rheological and textural properties of texture-modified diets for individuals with swallowing and mastication disorders. Significant variations in viscosity, maximum force, cohesiveness, and adhesiveness were observed among the ten thick purees analyzed despite all purees being within Level C of the BDA qualitative description. Oral processing had a notable impact on reducing viscosity, maximum force, and adhesiveness, while cohesiveness remained relatively unchanged. We suggest implementing standardized assessment protocols and instrumental quality control measuring viscosity using SI units to ensure consistent and safe texture-modified diets for individuals with dysphagia. Improving the standardization and quality control processes will enhance patient safety and improve outcomes in the management of patients with swallowing disorders. Future clinical trials should assess the therapeutic effects of these purees on the safety and efficacy of swallow by patients with dysphagia.

## Figures and Tables

**Table 1:** Characteristics and texture check for the thick puree (Level C) according to BDA qualitative classification.

Texture/Consistency Check	Texture/Consistency Characteristics
Thick puree should retain shape when scooped; Thick puree should be able to be held and eaten with a fork; A fork should create a distinct texture on the thick puree; Thick puree should not be too thin or spread easily; Thick puree should be thick enough to hold a spoon upright; Thick puree should not pass through piping.	Thick pureed texture and consistency; Smooth and easy to swallow, no chewing required; Fine texture, cohesive in the mouth; Consistent thickness throughout; Does not stick to the mouth or leave a residue; No decorations, hard pieces, or clusters; No separated liquids; No particles or hard pieces in the thick puree; Must not contain any kind of hard pieces or clusters that may form during the cooking process.

**Table 2:** An estimation of the ingredient’s percentage, weight in g for recipe, carbohydrate, protein, and number of kilocalories for all the thick purees tested. The Nautili Clear (xanthan gum-based) thickener% is referred to as weight in g.

Puree	Ingredients	Weight/Recipe (g)	Carbohydrates (g)	Proteins (g)	Kcal
French omelet	French omelet (86.2%), water (11.3%), thickener (1.4%), virgin olive oil (1.1%)	100	0.29	7.17	156.56
Zucchini omelet	Zucchini (44.6%), Ratatouille (8.9%), salt (0.2%), virgin olive oil (1.8%), diced potatoes (26.7%), water (17.8%), thickener (1.2%)	100	0.39	2.90	103.62
Pumpkin	Carrot (80.4%), Ratatouille (16.1%), salt (0.3%), virgin olive oil (3.2%), thickener (2%)	100	0.21	1.18	101
Stewed turkey	Sliced turkey breast (38.7%), salt (2.1%), virgin olive oil (8.82%), black pepper (0.8%), thickener (1.1%), water (32.5%), concentrated chicken broth without salt (14.8%), vegetable sauce (32.5%), diced potato, (22.3%), diced carrot (15.2%)	100	0.5	13.41	151.15
Pollock fish	Pollock fish (34.5%), salt (0.33%), olive oil (1.56%), black pepper (0.11%), thickener (0.22%), lemon sauce (62.28%)	100	-	10.21	113.18
Red lentils	Lentil (15.6%), Ratatouille (31.3%), concentrated chicken broth (3.9%), salt (0.1%), extra virgin oil (2%), diced potato (18.4%), diced carrot (7.8%), water (19.6%), ground cumin (0.2%), thickener (1.2%)	100	12.57	6.93	134.76
Noodles	Noodles nº2 (18.7%), olive oil (1.9%), salt (0.9%), Ratatouille base (9.3%), stock (55%), peeled shrimp (5.6%), garlic and parsley (1.9%), mussels (5.6), thickener (1.1%)	100	13	4.9	141.81
Hake fish	Hake fish (94.2%), salt (0.9%), olive oil (3.8%), black paper (0.4%) and thickener (0.7%)	100	-	10.19	113.1
Cauliflower	Cauliflower (44.6%), Ratatouille (8.9%), salt (0.2%), virgin oil (1.8%), diced potato (24.7%), water (17.8%) and thickener (2%)	100	5.3	2.75	74.48
Broccoli	Broccoli (44.6%), Ratatouille (8.9%), salt (0.2%), virgin oil (1.8%), diced potato (24.7%), water (17.8%) and thickener (2%)	100	6	2.75	74.48

**Table 3:** Viscosity values of the thick purees at 50 s^−1^ and percentage of reduction due to oral processing.% Show the reduction percentage due to the oral processing. The *p*-value shows significance between the pre- and post-oral processing.

Thick Puree	Viscosity (mPa·s)		% Decay Oral Processing 50 s^−1^	*p*-Value * by Oral Processing Effect 50 s^−1^
	Mean 50 s^−1^ (SD) Pre-Oral Processing	Mean 50 s^−1^ (SD) Post-Oral Processing		
French omelet	1915.42 ^c^ (268.95)	555.73 ^e^ (356.50)	70.99	<0.0001
Zucchini omelet	3111.48 ^b^ (1420.37)	1651.77 ^ab^ (687.51)	46.69	0.017
Pumpkin	1345.68 ^def^ (159.69)	1133.51 ^cd^ (209.5)	15.77	0.009
Stewed turkey	2093.76 ^c^ (414.48)	1063.52 ^cd^ (4341)	49.20	<0.0001
Pollock fish	1731.91 ^cd^ (201.66)	1575.82 ^ab^ (197.0)	9.01	0.04
Red lentils	1836.73 ^cd^ (488.62)	1055.02 ^d^ (404.81)	42.56	0.0001
Noodles	4809.85 ^a^ (1618.21)	1872.71 ^a^ (944.2)	61.07	<0.0001
Hake fish	1626.72 ^cde^ (248.26)	1389.90 ^bc^ (251.24)	14.55	0.04
Cauliflower	875.97 ^f^ (128.24)	862.91 ^de^ (122.75)	1.49	0.96
Broccoli	1095.34 ^ef^ (171.63)	1019.11 ^d^ (198.8)	6.96	0.25

Different superscript letters in the same column indicate significant differences (*p* < 0.05) according to Tukey’s test. * Mann–Whitney test between the pre-oral and post-oral processing.

**Table 4:** Viscosity values at 300 s^−1^ and percentage effect of oral processing on rheological properties. *p*-value (pre-post viscosity) shows significance between the pre- and post-oral processing.% shear thinning shows a reduction difference due to shear thinning between the shear rate 50 s^−1^ and the 300 s^−1^ and of shear rate. *p*-value ** (shear thinning) shows the significant differences between both shear thinning obtained at pre and post-oral processing.%, the reduction percentage due to the oral processing.

Thick Puree	Viscosity (mPa·s)		*p*-Value * (Pre-Post Viscosity)	Pre-Oral Shear Thinning%	*p*-Value ** (Shear Thinning)	% Effect on the Viscosity of Oral Processing Shear Thinning
	Mean 300 s^−1^ (SD) Pre-Oral Processing	Mean 300 s^−1^ (SD) Post-Oral Processing				
French omelet	518.48 ^c^ (126.89)	133.23 ^e^ (73.25)	<0.0001	72.93	0.79	93.04
Zucchini omelet	704.11 ^b^ (323.05)	388.96 ^ab^ (173.57)	0.014	77.37	0.42	87.44
Pumpkin	305.20 ^ef^ (39.49)	250.92 ^cd^ (46.96)	0.006	77.32	0.47	81.35
Stewed turkey	483.60 ^cd^ (101.94)	248.84 ^cd^ (86.70)	<0.0001	76.90	0.46	88.11
Pollock fish	354.04 ^de^ (31.60)	341.09 ^b^ (44.82)	0.031	79.55	0.02	79.87
Red lentils	464.22 ^cd^ (134.42)	252.23 ^cd^ (77.67)	0.0001	74.73	0.36	86.26
Noodles	1282.18 ^a^ (438.4)	458.55 ^a^ (241.70)	<0.0001	73.34	<0.0001	90.46
Hake fish	377.74 ^de^ (67.93)	317.40 ^bc^ (55.98)	0.014	76.77	0.32	80.48
Cauliflower	188.81 ^f^ (29.51)	280.81 ^de^ (38.84)	0.65	78.45	0.49	67.40
Broccoli	220.04 ^f^ (56.67)	221.5 ^d^ (45.84)	0.780	79.91	0.16	79.77

Different superscript letters in the same column indicate significant differences (*p* < 0.05) according to Tukey’s test. * Mann–Whitney test between the pre-oral and post-oral processing. ** Z test between the pre-oral and post-oral shear thinning.

**Table 5:** Flow index (n) and consistency index (k) mean values for all the thick purees at pre- and post-oral processing according to the Ostwald–de Waele equation.

Puree	Pre-Oral Processing		Post-Oral Processing	
	Flow Index (n)	Consistency Index	Flow Index (n)	Consistency Index
		(K; Pa⋅s^n^)		(K; Pa⋅s^n^)
French omelet	0.27	4.53	0.31	3.98
Zucchini omelet	0.27	4.63	0.93	4.57
Pumpkin	0.28	4.31	0.29	4.23
Stewed turkey	0.25	4.53	0.25	4.31
Pollock fish	0.22	4.52	0.24	4.48
Red lentils	0.87	3.27	0.06	4.82
Noodles	0.29	4.78	0.29	4.39
Hake fish	0.23	4.51	0.2	4.53
Cauliflower	0.28	4.14	0.26	4.15
Broccoli	0.28	4.21	0.27	4.2

**Table 6:** Maximum force and percentage effect of oral processing on textural properties with the *p*-value of comparing the pre- and post-oral processing of thick purees. *p*-value (pre-post maximum force) shows significance between the pre- and post-oral processing.% reduction percentage due to oral processing.

Thick Puree	Maximum Force (N) Pre-Oral Processing	Maximum Force (N) Post-Oral Processing	*p*-Value *	% Decay by Oral Processing
	Mean (SD)	Mean (SD)		
French omelet	0.74 ^b^ (0.24)	0.47 ^b^ (0.13)	<0.0001	36.49
Zucchini omelet	1.2 ^a^ (0.85)	0.85 ^a^ (0.52)	0.25	29.17
Pumpkin	0.52 ^b^ (0.11)	0.48 ^b^ (0.11)	0.08	7.69
Stewed turkey	0.61 ^b^ (0.11)	0.43 ^b^ (0.07)	0.0001	29.51
Pollock fish	0.64 ^b^ (0.13)	0.56 ^b^ (0.1)	0.093	9.38
Red lentils	0.51 ^b^ (0.09)	0.42 ^b^ (0.07)	0.003	17.65
Noodles	0.73 ^b^ (0.31)	0.52 ^b^ (0.11)	0.018	28.77
Hake fish	0.47 ^b^ (0.03)	0.44 ^b^ (0.05)	0.036	6.38
Cauliflower	0.5 ^b^ (0.06)	0.42 ^b^ (0.07)	0.08	16
Broccoli	0.53 ^b^ (0.11)	0.46 ^b^ (0.08)	0.013	13.21

Different superscript letters in the same column indicate significant differences (*p* < 0.05) according to Tukey’s test. * Mann–Whitney test between the pre-oral and post-oral processing.

**Table 7:** Cohesiveness and percentage effect of oral processing on textural properties with the *p*-value of comparing the pre- and post-oral processing of thick purees. *p*-value (pre-post cohesiveness) shows significance between the pre- and post-oral processing.% reduction percentage due to the oral processing.

Thick Puree	Cohesiveness Pre-Oral Processing	Cohesiveness Post-Oral Processing	*p*-Value *	% Decay by Oral Processing
	Mean (SD)	Mean (SD)		
French omelet	0.78 ^ab^ (0.08)	0.77 ^ab^ (0.05)	0.3	1.28
Zucchini omelet	0.66 ^c^ (0.08)	0.70 ^c^ (0.04)	0.1	−6.06 (increase)
Pumpkin	0.76 ^ab^ (0.03)	0.76 ^ab^ (0.04)	0.46	2.63
Stewed turkey	0.76 ^ab^ (0.05)	0.75 ^abc^ (0.01)	0.34	1.32
Pollock fish	0.78 ^ab^ (0.05)	0.74 ^abc^ (0.03)	0.021	3.9
Red lentils	0.79 ^ab^ (0.03)	0.76 ^ab^ (0.02)	0.023	3.8
Noodles	0.73 ^bc^ (0.15)	0.73 ^bc^ (0.09)	0.74	0
Hake fish	0.82 ^a^ (0.03)	0.78 ^ab^ (0.02)	0.001	4.88
Cauliflower	0.81 ^ab^ (0.04)	0.80 ^a^ (0.03)	0.098	1.23
Broccoli	0.82 ^a^ (0.04)	0.78 ^ab^ (0.03)	0.003	2.44

Different superscript letters in the same column indicate significant differences (*p* < 0.05) according to Tukey’s test. * Mann–Whitney test between the pre-oral and post-oral processing.

**Table 8:** Adhesiveness and percentage effect of oral processing on textural properties with the *p*-value of comparing the pre- and post-oral processing of thick purees. *p*-value (pre-post adhesiveness) shows significance between the pre- and post-oral processing.% reduction percentage due to oral processing.

Thick Puree	Adhesiveness (N·s) Pre-Oral Processing	Adhesiveness (N·s) Post-Oral Processing	*p*-Value *	% Decay by Oral Processing
	Mean (SD)	Mean (SD)		
French omelet	1.1 ^a^ (0.23)	0.54 ^ab^ (0.21)	<0.0001	50.91
Zucchini omelet	0.83 ^ab^ (0.51)	0.84 ^a^ (0.43)	0.74	−1.20 (increase)
Pumpkin	0.74 ^b^ (0.21) ^ab^	0.61 ^ab^ (0.17)	0.17	17.57
Stewed turkey	0.89 ^ab^ (0.027)	0.46 ^b^ (0.26)	<0.0001	48.31
Pollock fish	0.96 ^ab^ (0.20)	0.70 ^ab^ (0.23)	0.002	25
Red lentils	0.79 ^ab^ (0.26)	0.5 ^b^ (0.22)	0.005	36.71
Noodles	0.94 ^ab^ (0.53)	0.68 ^ab^ (0.38)	0.23	27.66
Hake fish	0.76 ^ab^ (0.07)	0.57 ^ab^ (0.13)	0.0001	25
Cauliflower	0.77 ^ab^ (0.20)	0.56 ^ab^ (0.28)	0.067	27.27
Broccoli	0.9 ^ab^ (0.23)	0.65 ^ab^ (0.25)	0.013	21.11

Different superscript letters in the same column indicate significant differences (*p* < 0.05) according to Tukey’s test. * Mann–Whitney test between the pre-oral and post-oral processing.

**Table 9:** Mean values of rheological and textural properties for ready-to-swallow thick purees. *p*-value (pre-post oral processing) shows significance between oral-processing sensitivity and oral-processing resistance.

SI Parameters	Oral-Processing Sensitive	Oral-Processing Resistant	*p*-Values *
	Mean (SD)	Mean (SD)	
Viscosity (mPa·s)	1287.24 (419.55)	941.01 (110.45)	0.001
Maximum force (N)	0.52 (0.14)	0.48 (0.07)	0.02
Cohesiveness	0.74 (0.02)	0.77 (0.03)	0.0001
Adhesiveness (N·s)	0.61 (0.12)	0.64 (0.07)	0.6

* Mann–Whitney test between the oral-processing sensitive and oral-processing resistant thick purees.

**Table 10:** Pearson correlation matrix coefficient values between different parameters, along with their corresponding *p*-values in parentheses at pre-oral processing.

SI Parameters	Viscosity Pre-Oral 300 s^−1^	Maximum Force Pre-Oral	Cohesiveness Pre-Oral	Adhesiveness Pre-Oral
Viscosity pre-oral 50 s^−1^ (*p*-value)	0.49 (<0.0001)	0.13 (0.11)	−0.25 (0.001)	−0.04 (0.59)
Viscosity pre-oral at 300 s^−1^ (*p*-value)		0.28 (0.0004)	−0.53 (<0.0001)	−0.09 (0.23)
Maximum force pre-oral (*p*-value)			−0.54 (<0.0001)	0.37 (<0.0001)
Cohesiveness pre-oral (*p*-value)				0.29 (0.0003)

A significant correlation was considered when (*p* < 0.05).

**Table 11:** Pearson correlation matrix coefficient values between different parameters, along with their corresponding *p*-values in parentheses at post-oral processing.

SI Parameters	Viscosity Post Oral 300 s^−1^	Maximum Force Post-Oral	Cohesiveness Post-Oral	Adhesiveness Post-Oral
Viscosity post-oral 50 s^−1^ (*p*-value)	0.47 (<0.0001)	0.29 (0.0003)	−0.42 (<0.0001)	−0.20 (0.013)
Viscosity Post-oral at 300 s^−1^ (*p*-value)		0.18 (0.14)	−0.50 (<0.0001)	−0.05 (0.65)
Maximum force post-oral (*p*-value)			−0.50 (<0.0001)	0.64 (<0.0001)
Cohesiveness post-oral (*p*-value)				−0.07 (0.34)

A significant correlation was considered when (*p* < 0.05).

## Data Availability

Not applicable.

## Supplementary Materials

The following supporting information can be downloaded at https://www.mdpi.com/article/10.3390/nu15173767/s1, Figure S1: Selected Mediterranean thick purees for this study.
