# Supplementary figures and images for: Measuring the Rheological and Textural Properties of Thick Purees Used to Manage Patients with Swallowing Disorders
Source: Nutrients. 2023 Aug 28;15(17):3767. doi: 10.3390/nu15173767 (PMC10490504; doi:10.3390/nu15173767)

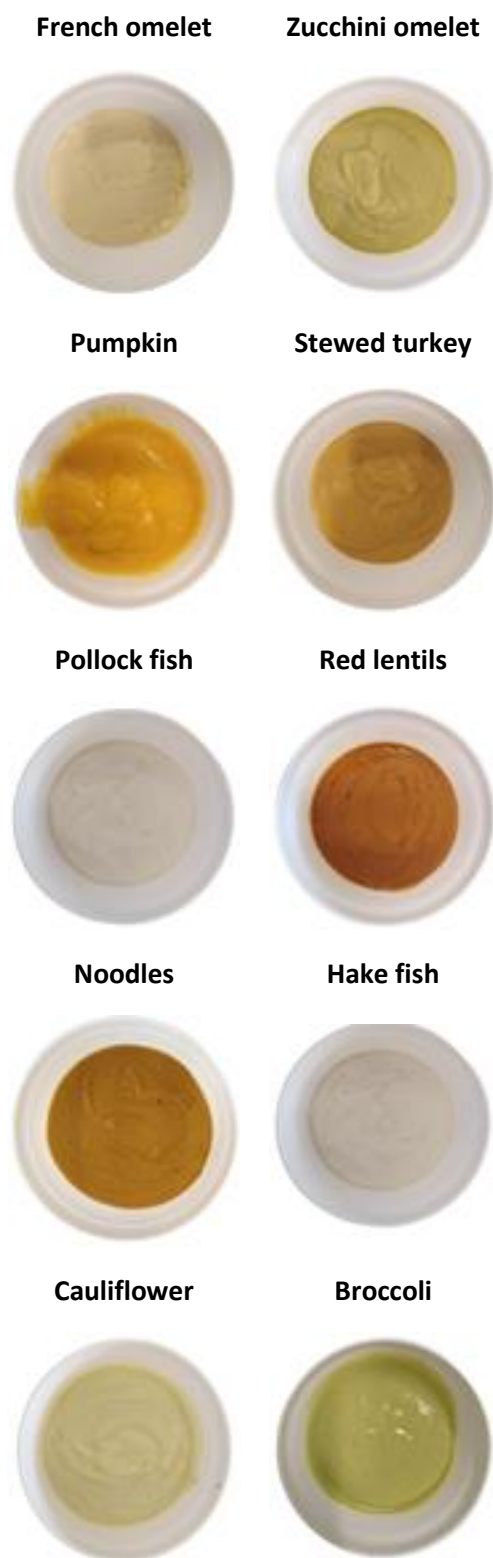

**Figure S1.** Selected Mediterranean thick puree for the study.

Supplement: Supplementary file 1 [file nutrients-15-03767-s001.zip › nutrients-2530216-supplementary.pdf]
